# Oral Brincidofovir Therapy for Monkeypox Outbreak: A Focused Review on the Therapeutic Potential, Clinical Studies, Patent Literature, and Prospects

**DOI:** 10.3390/biomedicines11020278

**Published:** 2023-01-19

**Authors:** Mohd. Imran, Mohammed Kanan Alshammari, Mandeep Kumar Arora, Amit Kumar Dubey, Sabya Sachi Das, Mehnaz Kamal, Abdulaziz Saad Abdulrahman Alqahtani, Mohammed Ahmed Yahya Sahloly, Ahmed Hammad Alshammari, Hessah Mohammed Alhomam, Aeshah Mousa Mahzari, Abida, Ali A. Rabaan, Tafadzwa Dzinamarira

**Affiliations:** 1 Department of Pharmaceutical Chemistry, Faculty of Pharmacy, Northern Border University, Rafha 91911, Saudi Arabia; 2 Department of Clinical Pharmacy, King Fahad Medical City, Riyadh 12211, Saudi Arabia; 3 School of Pharmaceutical and Population Health Informatics, DIT University, Dehradun 248009, Uttarakhand, India; 4 Department of Pharmaceutical Chemistry, College of Pharmacy, Prince Sattam Bin Abdulaziz University, Al-Kharj 11942, Saudi Arabia; 5 Department of Pharmaceutical Care, Prince Sultan Military Medical City, Riyadh 12233, Saudi Arabia; 6 Inventory Control Department, Rafha Maternity and Children Hospital, Rafha 76312, Saudi Arabia; 7 College of Clinical Pharmacy, King Faisal University, Al-Hofuf 31982, Saudi Arabia; 8 Al-Amal Pharmacy, Jazan 45142, Saudi Arabia; 9 Molecular Diagnostic Laboratory, Johns Hopkins Aramco Healthcare, Dhahran 31311, Saudi Arabia; 10 College of Medicine, Alfaisal University, Riyadh 11533, Saudi Arabia; 11 Department of Public Health and Nutrition, The University of Haripur, Haripur 22610, Pakistan; 12 ICAP, Columbia University, Harare P.O. Box 28, Zimbabwe; 13 School of Health Systems and Public Health, University of Pretoria, Pretoria 0002, South Africa

**Keywords:** brincidofovir, CMX001, cidofovir, monkeypox, smallpox, Orthopoxvirus, patent

## Abstract

The monkeypox disease (MPX) outbreak of 2022 has been reported in more than one hundred countries and is becoming a global concern. Unfortunately, only a few treatments, such as tecovirimat (TCV), are available against MPX. Brincidofovir (BCV) is a United States Food and Drug Administration (USFDA)-approved antiviral against smallpox. This article reviews the potential of BCV for treating MPX and other Orthopoxvirus (OPXVs) diseases. The literature for this review was collected from PubMed, authentic websites (USFDA, Chimerix), and freely available patent databases (USPTO, Espacenet, and Patentscope). BCV (a lipophilic derivative of cidofovir) has been discovered and developed by Chimerix Incorporation, USA. Besides smallpox, BCV has also been tested clinically for various viral infections (adenovirus, cytomegalovirus, ebola virus, herpes simplex virus, and double-stranded DNA virus). Many health agencies and reports have recommended using BCV for MPX. However, no health agency has yet approved BCV for MPX. Accordingly, the off-label use of BCV is anticipated for MPX and various viral diseases. The patent literature revealed some important antiviral compositions of BCV. The authors believe there is a huge opportunity to create novel, inventive, and patentable BCV-based antiviral therapies (new combinations with existing antivirals) for OPXVs illnesses (MPX, smallpox, cowpox, camelpox, and vaccinia). It is also advised to conduct drug interaction (food, drug, and disease interaction) and drug resistance investigations on BCV while developing its combinations with other medications. The BCV-based drug repurposing options are also open for further exploration. BCV offers a promising opportunity for biosecurity against OPXV-based bioterrorism attacks and to control the MPX outbreak of 2022.

## 1. Introduction

The genus Orthopoxvirus (OPXV), belonging to the family Poxviridae, includes major human pathogens such as variola virus (VARV), monkeypox virus (MPXV), cowpox virus (CPXV), camelpox virus (CMLV), and vaccinia virus (VACV) [1,2]. The main concern of 2022 was the monkeypox disease (MPX) outbreak, which is spread by MPXV (double-stranded DNA virus). Different species of rodents are considered the primary natural reservoir of zoonotic MPXV [1,2,3]. As of 19 October 2022, MPX cases were reported in 109 countries of different regions (Africa, America, Eastern Mediterranean, Europe, South-East Asia, and Western Pacific), with 73,437 confirmed cases and 29 deaths [4]. MPXV was first identified in 1958 in monkeys, but the first MPX in a human case was reported in 1970 [3]. MPX outbreaks have also been reported in the past (from 1997 to 2020) in the African region, Singapore, the United Kingdom, and the United States of America [3]. However, the MPX outbreak of 2022 is serious because it is spreading in a larger part of the world compared to past episodes. MPX transmission is possible when a normal person comes in contact with the skin lesion, respiratory droplet, and body fluid of the infected animal/human. MPX transmission is also possible through an infected sexual partner [5]. The MPXV enters the body, replicates at the infection site, causes viremia, infects the lymph nodes, and produces symptoms [3,6]. The clinical signs of MPX appear within 5–21 days after MPXV infection and comprise fever, headache, chills, rashes in different body parts, myalgia, asthenia, and lymphadenopathy [3,7]. In addition, various complications related to MPX are also reported, including encephalitis, pneumonitis, minor bacterial infections, and sight-menacing keratitis [8,9,10]. MPXV has two clades: the West African clade (WAC) and Congo Basin or Central African clade (CAC). The CAC is believed to be more transmissible and virulent than WAC. It is thought that MPXV infection in non-African countries is due to the WAC [5,11,12]. MPX and smallpox share common signs and symptoms, creating difficulties in diagnosing MPX. Sequencing analysis and PCR tests can be used to diagnose MPX. However, MPX’s lymphadenopathy (prodromal stage) is an important feature of MPX, which differentiates MPX from smallpox [7,13]. The untreated MPX can trigger sepsis, pneumonitis, sight-menacing keratitis, encephalitis, loss of sight, and death (up to 6%) [8,9,10,14]. Remission of smallpox vaccination, rising human–animal interaction, enhanced commute to affected regions, lessened international co-ordination, complexity in diagnosing MPX, lack of MPX studies, and availability of a few treatment options (tecovirimat) are the factors promoting the MPX spread across the globe [3,15].

Brincidofovir (BCV) is a United States Food and Drug Administration (USFDA)-approved antiviral against smallpox. Smallpox is caused by VARV, which shares many features with MPXV. Accordingly, various health agencies have recommended using BCV for MPX. However, no health agency has yet approved BCV for MPX. This article reviews the potential of BCV for MPX and other OPXVs infections. The literature for this review was collected from PubMed, authentic websites (USFDA, Chimerix, and clinicaltrial.gov), and freely available patent databases (USPTO, Espacenet, and Patentscope).

## 2. Brincidofovir (BCV)

### 2.1. Chemistry

**Figure 1:**
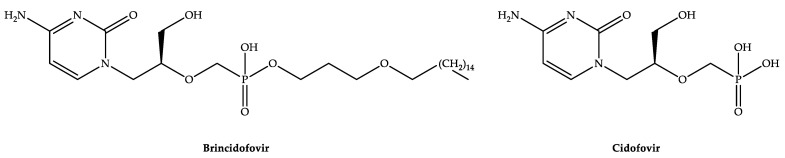
Chemical structures of BCV and CDV.

Brincidofovir (synonym: BCV, CMX001, Hexadecyloxypropyl-cidofovir, and HDP-cidofovir; CAS registry number: 444805-28-1; chemical name: Phosphonic acid, P-[[(1S)-2-(4-amino-2-oxo-1(2H)-pyrimidinyl)-1-(hydroxymethyl)ethoxy]methyl]-, mono [3-(hexadecyloxy)propyl] ester; molecular formula: C_27_H_52_N_3_O_7_P; molecular weight: 561.70) (Figure 1) is a covalently linked lipid conjugate (phosphonate ester prodrug) of cidofovir (CDV) (Figure 1) [16,17,18,19,20].

BCV has been developed by Chimerix [16,21]. BCV is a water-insoluble white/off-white crystalline solid classified as a BCS IV drug substance (low solubility and permeability). BCV is a free acid and exhibits pH-dependent solubility and dissolution (BCV solubility increases as pH increases) [16]. BCV is a novel lipid conjugate of CDV, but it was not designated as a new molecule entity (NME) by the USFDA because its active moiety is CDV, which was approved by the USFDA in 1996 [16].

### 2.2. Regulatory Approval

In June 2021, the USFDA approved oral BCV for treating human smallpox disease under the Animal Rule (Table 1) [21,22].

**Figure 2:**
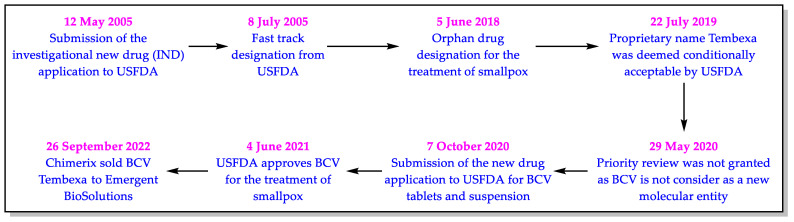
USFDA regulatory development cycle of BCV.

When writing this article, BCV is only approved by the USFDA for treating smallpox. The regulatory development cycle of BCV at the USFDA is provided in Figure 2.

BCV has not been approved by the USFDA or any other health agency to treat MPX. However, the Center for Disease Control (CDC) and the literature have recommended using BCV to treat MPXV disease [12,13,23,24,25].

### 2.3. Pharmacology of BCV

#### 2.3.1. In Vitro Anti-OPXV Activity of BCV

BCV has demonstrated appreciable and consistent anti-OPXV virus activity in cell cultures [26,27,28,29,30]. The summary of the in vitro activity of BCV against different OPXVs is provided in Table 2.

#### 2.3.2. Efficacy of BCV in Animal Model

The BCV efficacy evaluation is not feasible in humans because of its devastating nature. Therefore, the efficacy of BCV for smallpox was established under Animal Rule with two relevant studies, the rabbit model (CMX001-VIR-106, rabbitpox model) and the mouse model (CMX001-VIR-044, ECTV model) [26,31,32]. The RPXV and ECTV models demonstrated a reduction in the mortality of the infected animals and confirmed the BCV’s efficacy for OPXV diseases (Table 3) [26,31,32].

#### 2.3.3. Mechanism of Action of BCV

**Figure 3:**
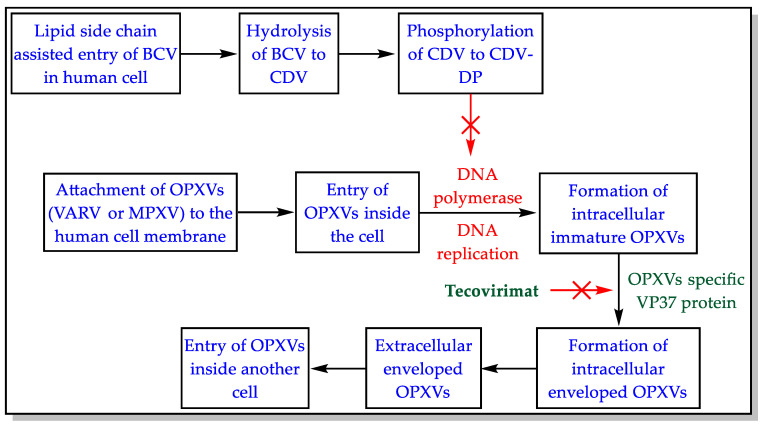
Mechanism of action of BCV.

BCV is a phosphonate ester prodrug of CDV (injectable drug) [16,20,23,33,34,35]. The lipid moiety of BCV governs the oral absorption, distribution, pharmacokinetic, and higher intracellular concentration of BCV. The lipophilic side chain of BCV mimics the natural phospholipid (lysophosphatidylcholine) and enters the cell utilizing endogenous lipid uptake pathways. The lipid side chain of BCV hydrolyzes in the cell to provide CDV, which is phosphorylated to CDV-diphosphate (CDV-DP). The CDV-DP inhibits the DNA polymerase enzyme that aids viral DNA synthesis. CDV-DP also acts as an alternate substrate (nucleotide analog) incorporated into the developing viral DNA string and slows down the DNA synthesis [16,20,23,33,34,35]. The OPXVs (VARV, MPXV, CPXV, CMLV, and VACV) share similar enzymes for the replication of their DNA [1,2,3]. Therefore, BCV is thought to inhibit MPXV, CPXV, CMLV, and VACV in addition to VARV. The mechanism of action of BCV against MPXV is provided in Figure 3.

BCV has an extended half-life due to CDV-DP. The lipid moiety increases the oral bioavailability of BCV and makes it a target drug delivery with reduced nephrotoxic side effects associated with CDV [16,34,35].

#### 2.3.4. Other Pharmacological Parameters of BCV

The important pharmacological parameters of BCV are summarized in Table 4 [16,32,33,34,35].

## 3. Clinical Studies (CSs) on BCV

A search on the CSs on BCV was conducted on the clinical trial database [36] on 28 October 2022, employing three keywords (Brincidofovir = 22 studies; CMX001= 22 studies; HDP-Cidofovir = 22 studies). The identical results were removed, and the remaining results were summarized (Table 5). The CSs have been performed by Chimerix, SymBio Pharmaceuticals, Assistance Publique, and the University of Alabama (Table 5). The CSs were related to viral infections/conditions (viruria, double-stranded-DNA virus (ds-DNAV), BK virus (BKV), adenovirus (AdV), ebola virus (EV), herpes simplex virus (HSV), and cytomegalovirus (CMV)), pharmacokinetic, and drug interactions of BCV (Table 5).

A PubMed search using filters (clinical trial; randomized clinical trial) was also carried out on 28 October 2022, utilizing the keywords (All NCT numbers mentioned in Table 5; NCT00942305 = 1 study; Brincidofovir = 8 studies; CMX001 = 9 studies; HDP-Cidofovir = none). The identical results of the search were removed. The deduplicated results are summarized (Table 6).

## 4. Patent Searching and Patent Summary

The patent searching was performed on the freely available patent databases (Espacenet, Patentscope, and USPTO) on 28 October 2022, utilizing different keywords (Brincidofovir, CMX001, HDP-Cidofovir, and Chimerix) and the combinations of these keywords [45,46,47,48,49]. The innovator/developer of BCV is Chimerix Inc. When the USFDA approves a drug, the innovator submits important patents related to the USFDA’s Orange Book (OB) database [50]. Therefore, all the patents/patent applications of BCV assigned to Chimerix Inc. and those listed in the OB of the USFDA have been included and summarized in this review. The patents/patent applications assigned to other applicants explicitly claiming the inventions of BCV have also been included and summarized in this review (Table 7).

## 5. Discussion

OPXVs-based bioterrorism is a concern because certain groups may own pathogenic OPXVs (such as VARV that causes smallpox) as undeclared items; the possibility of creating OPXVs utilizing a synthetic biology approach; and development of drug resistance against the available treatment of OPXVs infections [18,20]. The current global population has inadequate immunity against VARV, MPXV, and other OPXVs [18]. Accordingly, the re-emergence of smallpox and the emergence of other OPXV infections (such as MPX) may be devastating. Therefore, USFDA prepared strategies to develop treatments (BCV and TCV) for VARV and other OPXV infections [34]. The spread of MPX was the global concern of 2022. The smallpox vaccine offers 85% protection versus MPX [71,72]. The cessation of smallpox vaccination is considered one factor in increasing MPX cases. This also highlights the severity of MPX in pediatric patients compared to adult patients [72].

CDV, a nephrotoxic antiviral with poor oral bioavailability, was discovered in 1986, and intravenous CDV was approved by USFDA in 1996 for CMV retinitis in AIDS patients [17]. Despite the nephrotoxicity, CDV was the first antiviral stockpiled for VARV re-emergence [17]. BCV is a USFDA-approved treatment for VARV infection (smallpox) [21,22]. BCV (patient compliant orally bioavailable, non-nephrotoxic/non-myelotoxic lipophilic prodrug of CDV) was created to increase the oral bioavailability of CDV, combat VARV bioterrorism strikes, and tackle complications associated with smallpox vaccination [51,56,71,73,74,75,76]. BCV and CDV have shown good in vitro activity against OPXVs (MPX, VARV, etc.), CMV, HSV, EBV, AdV, BK virus, JC virus, human papillomavirus, and other ds-DNAVs [18,23,73,76]. The in vitro activity of BCV is better than CDV against OPXVs (VARV, MPXV, etc.) [17]. Different animal models of MPX exhibited promising efficacy of BCV, but BCV’s effectiveness is not fully established in humans against MPXV [12,17,19,23,34,71,77]. CDC and the literature have recommended using BCV to treat MPXV disease [12,13,23,24,25,78]. One study has endorsed the immediate use of BCV to treat individuals exposed to MPXV [78]. Further, VARV and MPX share common genetic makeup. Therefore, drugs (BCV and TCV) used for VARV infection (smallpox) may be effective against MPX [1,2]. Accordingly, BCV has the potential to battle the challenge of the MPX outbreak of 2022.

Drug resistance is an important factor in the success of treatment. Resistance to CDV due to mutation in OPXVs specific DNA polymerases is documented [17,71]. Genetic variations in MPXV clades are also reported. These clades differ from each other concerning their severity and mortality [72]. The resistance to BCV and CDV-DP is also imaginable [75]. There is a high possibility that MPXV may further mutate, necessitating new containment and treatment strategies [19]. Therefore, BCV’s effectiveness against a different variant of MPX needs investigation. TCV is an approved treatment for MPX [3]. The mechanisms of action of TCV and BCV are distinct (Figure 3). Resistance to BCV and TCV monotherapy is possible. However, combining BCV and TCV will be of great use to combat MPX and avoid drug resistance issues [20,34].

Drug repurposing for BCV is a foreseeable area of research. Reports have suggested repurposing BCV for COVID-19 [79]. Drug repurposing studies against various viral diseases have also been planned for BCV by Symbio [75]. The non-nephrotoxic/non-myelotoxic nature of BCV and its effectiveness against multiple ds-DNAVs makes it a suitable candidate for drug repurposing for many viral diseases treated by nephrotoxic and myelotoxic antivirals (CDV, foscarnet, ganciclovir, etc.) [75]. Accordingly, BCV may be approved by global health agencies for different viral diseases.

BCV is not nephrotoxic because it does not accumulate in the kidney [17]. However, BCV can elevate the transaminases in the liver [12,17,23,74]. Therefore, BCV treatment in hepato-compromised individuals should be monitored and accompanied by liver function tests. Cyclosporine (inhibitor of OATP1B1 and OATP1B3) augments the concentration of BCV [75]. BCV is an acidic drug and can interact with basic medications. Therefore, the interaction of BCV with OATP1B1/OATP1B3 inhibitors and basic drugs needs consideration during BCV therapy. Smallpox treatment with BCV involves a weekly dose of 200 mg for two weeks (Table 4). The BCV therapy (oral 200 mg/week) was discontinued in a case study due to elevated liver enzymes [12]. This observation hints at some foreseeable challenges to clinicians regarding the starting time, optimal dose selection, and the duration of BCV therapy for MPX. Data are scarce on BCV’s cellular toxicity and special populations (pregnant women, lactating mothers, geriatric patients, etc.) [17,71,75]. This aspect is another factor that needs to be taken care of. ASM converts BCV to CDV (Table 4). In certain patients, the content of ASM may be low. Therefore, screening of such patients is recommended before BCV therapy.

The development of innovations is based on the knowledge of the existing patent and nonpatent literature. Accordingly, the authors carried out a patent literature search on BCV. Chimerix is the owner of some patents related to BCV (Table 7). These patents claim different polymorph, synthetic methods to prepare BCV, and compositions of BCV for treating viral diseases, including MPX, CMV, AdV, and EBV [51,52,53,54,55,56,57,58,59,60,61]. BCV has gastrointestinal toxicity (nausea, vomiting, and diarrhea). This side effect is because of the high concentration of BCV in the small intestine [75]. The sodium salt (inorganic salt) of BCV is reported in the patent literature [51]. The development of IV dosage from the sodium salt of BCV may reduce the gastrointestinal toxicity of oral BCV [17,75]. The organic salt of a drug has a lipophilic characteristic and is suitable for developing topical dosage forms (ointment, gels, creams, etc.) [50]. MPX is characterized by skin lesions, which may be partially or fully treated with topical dosage forms. Accordingly, patenting of lipophilic salts of BCV by various pharmaceutical companies is foreseeable. The oral bioavailability and safety profile of BCV is better than CDV (nephrotoxic). Therefore, some important inventions have claimed improved treatment outcomes of antiviral therapy by replacing CVD and ganciclovir with BCV [62,63,64,65,66,67,68,69,70]. The authors predict the development of further BCV-based antiviral combinations with good safety profiles against OPXV infections, including MPX.

The drug utilized to treat a viral outbreak must be potent, have a shorter therapy duration, be patient-compliant, should not pose a risk of resistance to highly replicating viruses, and must have the least drug interactions [18]. BCV possesses most of these features, making it a suitable candidate to combat OPXVs-induced outbreaks, including MPX.

## 6. Conclusions

BCV is only approved in the United States for smallpox. Various health agencies and clinical case reports have endorsed the off-label use of BCV as an option for MPX. BCV can potentially combat MPX and biosecurity weapons against OPXVs-related bioterrorism strikes. The mutations in OPXVs may cause BCV resistance. Combining BCV with TCV may be an option to tackle the BCV/TCV resistance issue. The authors recommend further BCV-based drug–drug, drug–food, and drug–disease interaction studies that may help to optimize BCV therapy. The BCV-based drug repurposing studies for different viral infections and drug combinations investigations with other antivirals are also foreseeable and recommended.

## Figures and Tables

**Figure 4:**
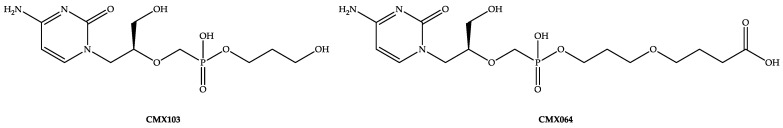
Chemical structures of inactive metabolites of BCV.

**Table 1:** Rx data of BCV.

Proprietary Name (Active Ingredient; Applicant; Marketing Status)	Dosage Form (Route of Administration)	Strength (Approval Date)	Exclusivity Data	Approved Indication
Tembexa (Brincidofovir; Chimerix Inc; Prescription)	Tablet (Oral)	100 mg (4 June 2021)	New product exclusivity expires on 4 June 2024; Orphan drug exclusivity expires on 4 June 2028	Treatment of human smallpox diseases caused by variola virus in adult and pediatric patients, including neonates
	Suspension (Oral)	10 mg/ mL (4 June 2021)		

**Table 2:** In vitro anti-OPXV activity of BCV.

OPXV	Strains	Cells	EC_50_ Range (µM)
VARV [26]	BSH74, SOM77, JAP51, UNK52, BRZ66, and BSH	BSC-40, Vero 76, and MK2	0.04–0.21
Rabbitpox virus (RPXV) [26]	Utrecht	BSC-40 and Vero 76	0.5–1.89
Ectromelia virus [26]	Moscow	BSC-40, CV-1, and BSC-1	0.125–0.5
VACV [26]	WR, Copenhagen, Lister, Elstree, and IHD	CV-1, HEL, HFF, Vero, C127I, and PHK	0.004–1.2
CPXV [26]	Brighton and five different isolates	HFF, HEL, and PHK	0.007–0.6
CMLV [26]	Iran (CML1)	HEL	0.021–0.024
MPXV [26]	No data	No data	0.023–0.12
VACV/RPXV chimera [26]	VACV-WR/RPXV E9L	BSC-40	1.75

**Table 3:** BCV’s efficacy in rabbitpox and mousepox model.

Model	Dose Regimen	Treatment Initiation Day	% Survival	
			Placebo	BCV
Rabbitpox [26,32]	20/5/5 mg/kg (fully effective dose in the rabbitpox model)	Day 4	29%	90%
Mousepox [26,32]	10/5/5 mg/kg (fully effective dose in the mousepox model)	Day 4	13%	78%

**Table 4:** Important pharmacological parameters of BCV [16,32,33,34,35].

Parameter	Summary
**Dose**	Maximum 200 mg/day for patients weighing ≥ 48 kg. BCV can be taken on an empty stomach or with a low-fat meal (about 400 calories, 25% fat). Healthy adults tolerated single doses of BCV up to 350 mg PO (tablet) and 50 mg IV and multiple doses (total of 4) of BCV up to 20 mg IV.
**Treatment duration**	Weekly dose for two weeks because CDV-DP has an exceedingly longer duration of action. No dose adjustment is necessary for patients with hepatic/renal impairment because treatment consists of only two doses (day one and day 8). The shorter duration also reduces the chances of adverse reactions.
**Absorption**	Oral bioavailability: 13.4% (Tablet) and 16.8% (suspension); C_max_ of BCV: 480 ng/mL; T_max_ of BCV: 3 h; absorption decreases with the fatty meal because BCV is an acidic drug.
**Volume of distribution**	1230 L
**Protein binding**	>99.9% bound to human plasma proteins
**Blood plasma ratio**	0.48 to 0.61 in healthy adults
**Metabolism**	Acid sphingomyelinase (ASM) is an important enzyme for the metabolism of BCV to CDV. The CYPF42 pathway converts BCV to inactive metabolites (CMX103 and CMX064) (Figure 4).
**Route of elimination**	The metabolites are excreted in urine (51%) and feces (49%). Unchanged BCV has not been detected in urine or feces.
**Half-life**	BCV: 19.3 h; CDV-DP: 113 h
**Clearance**	44.1 L/h
**Adverse Effects**	Diarrhea; abdominal pain; nausea; vomiting; elevation in hepatic transaminase/bilirubin; toxic to embryo-fetus; human carcinogen; male infertility.
**Drug interaction**	BCV is a substrate of OATP1B1 or 1B3 membrane uptake transporters. BCV exposures can be increased by the concomitant use of OATP1B1 or 1B3 inhibitors. Coadministration of BCV with a proton pump inhibitor could theoretically increase either the absorption rate or the extent of absorption.
**Contraindication**	Avoid BCV coadministration with CDV
**Toxicity/Overdose**	No clinical case of toxicity/overdose was reported.
**QT prolongation**	No significant QTc prolongation effect of BCV (200 mg) was detected in a TQT study using moxifloxacin (400 mg) as the positive control.

**Table 5:** Summation of the CSs on BCV provided in the clinical trial database [36].

NCT Numbers (Sponsor; Location)	Condition/Disease (Intervention)	Assessment	Phase (Study Type; Purpose; Number Enrolled)	Status (Completion Date; Last Update on the Database)
**NCT02087306** (Chimerix; United States)	AdV (BCV)	Safety and efficacy	3 (Interventional; Treatment; 201)	Completed (August 2016; 13 August 2021)
**NCT01143181** (Chimerix; United States)	ds-DNAV (BCV)	Treatment	3 (Interventional; Treatment; 210)	Completed (December 2012; 12 August 2021)
**NCT01241344** (Chimerix; United States)	AdV (BCV; Placebo)	Safety and efficacy	2 (Interventional; Treatment; 52)	Completed (June 2013; 21 July 2021)
**NCT00793598** (Chimerix; United States)	BKV viruria (BCV; Placebo)	Treatment	1 and 2 (Interventional; Treatment; 29)	Completed (October 2010; 16 August 2021)
**NCT00942305** (Chimerix; United States)	CMV (BCV; Placebo)	Prevention/control	2 (Interventional; Prevention; 239)	Completed (January 2012; 16 July 2021)
**NCT01769170** (Chimerix; United States)	CMV (BCV; Placebo)	Safety and efficacy	3 (Interventional; Treatment; 452)	Completed (January 2016; 21 July 2021)
**NCT00780182** (Chimerix and NIH; United States)	Healthy subjects (BCV)	Effect of high-fat food on the bioavailability	1 (Interventional; Treatment; 24)	Completed (January 2009; 2 February 2010)
**NCT04542252** (SymBio Pharmaceuticals; Japan)	Drug–drug interaction (BCV; cyclosporine; SyB V-1901)	Pharmacokinetic	1 (Interventional; Drug interaction; 13)	Completed (January 29, 2021; 27 April 2021)
**NCT05391724** (Chimerix; United States)	Hepatic impairment (BCV)	Safety and pharmacokinetic	1 (Interventional; Not mentioned; 25)	Completed (September 2011; 26 May 2022)
**NCT05511779** (SymBio Pharmaceuticals; Australia)	BKV, nephropathy, and kidney transplantation (BCV)	Safety and tolerability	2 (Interventional; Treatment; 36)	Not yet recruiting (Not yet recruiting; 23 August 2022)
**NCT04706923** (SymBio Pharmaceuticals; United States)	AdV (BCV)	Safety and tolerability of IV BCV	2 (Interventional; Treatment; 24)	Recruiting (Recruiting; 31 May 2022)
**NCT03481244** (Assistance Publique; France)	AdV (BCV; CCDV)	Efficacy and toxicity	Not mentioned (Observational; Prospective; 400)	Not yet recruiting (Not yet recruiting; 10 February 2020)
**NCT02439957** (Chimerix; United States)	CMV and kidney transplant infection (BCV; Valganciclovir)	Safety and efficacy of BCV versus valganciclovir	3 (Interventional; Prevention; 6)	Terminated (Terminated; 16 July 2021)
**NCT02439970** (Chimerix; United States)	CMV (BCV; Valganciclovir)	Safety and efficacy	3 (Interventional; Prevention; 5)	Terminated (Terminated; 16 July 2021)
**NCT03339401** (Chimerix; United States)	AdV (Standard of care (Soc); BCV)	Safety and efficacy	2 (Interventional; Treatment; 29)	Terminated (Terminated; 25 January 2021)
**NCT02420080** (Chimerix; United States)	AdV (BCV)	To obtain retrospective data	Not mentioned (Observational; Determine rates of AdV progression and mortality; 100)	Terminated (Terminated; 9 January 2017)
**NCT02167685** (Chimerix; United States)	Outcomes and survival rates (BCV)	To establish a registry database	Not mentioned (Observational; Prospective; 550)	Terminated (Terminated; 17 May 2019)
**NCT01610765** (University of Alabama; United States)	Herpes simplex virus (BCV; Placebo)	Safety and dose determination	1 and 2 (Interventional; Treatment; zero)	Withdrawn (Withdrawn; 7 June 2016)
**NCT04268966** (Chimerix and USFDA; United States)	EV (BCV)	Safety and tolerability	2 (Interventional; Treatment; zero)	Withdrawn (Withdrawn; 26 February 2020)
**NCT02271347** (Chimerix; Not mentioned)	EV (BCV)	Safety and efficacy	2 (Interventional; Treatment; Zero)	Withdrawn (Withdrawn; 2 February 2015)
**NCT03532035** (Chimerix; United States)	AdV (BCV; SoC)	Safety, tolerability, and pharmacokinetic	2 (Interventional; Treatment; Zero)	Withdrawn (Withdrawn; 21 July 2021)
**NCT02596997** (Chimerix; United States)	AdV (BCV)	Treatment	Not mentioned (Expanded access; Treatment; Not mentioned)	No longer available (Not available; 14 March 2022)

**Table 6:** Summation of the PubMed-published CSs on BCV.

Year [Ref. No.]	Summary
2019 [37]	This study is related to NCT01769170 and evaluated oral BCV for the prevention of CMV infection in allogeneic hematopoietic cell transplant (HCT) patients. BCV did not demonstrate a lowering in CMV infections, even after 24 weeks and showed gastrointestinal toxicity.
2017 [38]	The study relates to the treatment of HCT-linked adenoviremia with BCV. BCV was highly efficacious and tolerable in controlling adenoviremia. Abdominal cramps and diarrhea were observed as side effects, but no nephrotoxicity was noticed.
2017 [39]	This study evaluated the BCV benefit-to-risk profile for treating smallpox. BCV was well tolerated in adults and children at doses and durations equivalent to smallpox treatment. Mild gastrointestinal problems and transitory, asymptomatic transaminase increases were most prevalent.
2017 [40]	This CS tested BCV for preventing AdV illness in HCT patients (pediatric/adult). This trial verified BCV’s anti-AdV activity. The most commonly observed side effect was diarrhea. Myelosuppression and nephrotoxicity were not noted.
2016 [41]	This CS focused on BCV’s efficacy against the EV. However, the CS was halted because the BCV’s manufacturer decided to stop working on it as a treatment for EV disease (EVD). No serious side effects were found. Because of the low sample size, it was impossible to determine if BCV worked to treat EVD.
2016 [42]	This study assessed the development of mutation and resistance in CMV’s gene involved in the production of CMV DNA polymerase. The results did not reveal mutation and CMV antiviral resistance among BCV-treated subjects.
2013 [43]	This study is related to NCT00942305 and assessed the safety and efficacy of BCV against CMV infection in allogeneic HCT patients. Patients who took BCV (100 mg two times per week) experienced fewer CMV episodes than those who received a placebo. The most commonly observed side effect was diarrhea. Myelosuppression and nephrotoxicity were not noted.
2012 [44]	The study evaluated BCV’s safety and pharmacokinetics after single and multiple doses. Blood chemistry, hematology, renal function, and intraocular pressure did not change clinically. There were no BCV-related mucosal alterations. Maximum plasma concentrations of BCV were seen 2 to 3 h post-dose. In healthy volunteers, BCV was well tolerated (2 mg/kg or 140 mg) in adults.

**Table 7:** Summary of the important patents/patent applications of BCV.

Patent/Application (Applicant/Status)	Summary of the Claims
**Patents/patent applications filed by Chimerix**	
**US9303051B2** (Patented case)	This OB-listed patent claims crystalline Form B of BCV. It also claims a pharmaceutical composition comprising BCV and a pharmaceutically acceptable carrier. It further claims the treatment of many viral diseases, including smallpox, MPX, cowpox, camelpox, and ebola using BCV. It also discloses the sodium salt of BCV [51].
**US8962829B1** (Patented case)	This OB-listed patent claims crystalline Form II of BCV and its preparation method. It also discloses Form H and some process-related impurities of BCV [52].
**US9371344B2** (Patented case)	This OB-listed patent claims a method of preparing Form II of BCV. It also claims a composition of Form II of BCV with compounds designated as compounds A, B, C, and D [53].
**US10487061B2** (Patented case)	This OB-listed patent claims a method of preventing/treating viral infections (a double-stranded DNA viral infection) using a composition of Form II of BCV and a pharmaceutically acceptable carrier. It also discloses an amorphous form of BCV [54].
**US10112909B2** (Patented case)	This OB-listed patent claims a method of preventing/treating viral infections (ds-DNA viral infection) using Form II of BCV [55].
**US8569321B2** (Patented case)	Crystalline Form A of BCV, its pharmaceutical composition, and method of preparation [56].
**US9862687B2** (Patented case)	A method of synthesizing a compound that can be used to synthesize BCV [57].
**US11066373B2** (Patented case)	A method of preparing crystalline Form II of BCV [58].
**WO2018156879A1** (No national phase entry)	The use of BCV in preventing/treating AdV infection in patients experiencing toxic side effects or nephrotoxicity due to other treatments (CDV, cyclic CDV, tenofovir, and adefovir) [59].
**US2017368082A1** (Abandoned)	A pharmaceutical composition having a pH of about 8, wherein the composition comprises BCV, a bulking agent, a buffer, and water. It also claims a lyophilized powder containing BCV, mannitol, and arginine. It further relates to using the claimed compositions to treat viral infections, including AdV, CMV, OPXVs, etc. [60].
**US2020138835A1** (Abandoned)	A lyophilized powder comprising BCV, mannitol, and arginine. It also claims a method of treating a viral infection (AdV, CMV, OPXVs, etc.) using the claimed lyophilized powder [61].
**Patents/patent applications filed by other companies**	
**US10828317B2** (Ultupharma; Patented case)	A composition comprising a nucleoside analog (gemcitabine, didanosine, 5-fluorouracil, CDV, BCV, etc.) capable of decreasing bacterial colonization or infection of a subject; a second compound (uridine or cytidine) capable of reducing mitochondrial toxicity and increasing the antibacterial effect of the nucleoside analog; and iclaprim capable of decreasing the concentration in bacteria of nucleosides and nucleotides known to compete with nucleoside analogs [62].
**US9562232B2** (University of Massachusetts; Patented case)	A method of inhibiting HCMV replication in a cell using a miR132 antagonist (antisense locked nucleic acid) alone or in combination with an antiviral agent (CDV, BCV, ganciclovir, valganciclovir, and acyclovir) [63].
**US10081670B2** (Regeneron Pharmaceuticals; Patented case)	A method of neutralizing infectious EBV utilizing anti-EBV antibodies or antigen-binding fragments alone or in combination with additional antiviral agents (BCV and favipiravir) [64].
**US11071745B2** (Elian; Patented case)	A method of preventing HSV infection employing a composition comprising valacyclovir and famciclovir, which may additionally include CDV or BCV [65].
**US2020072848A1** (University of Miami; Under examination)	A prophylactic method for an immune-compromised patient (organ transplant or cancer therapy patient) with a high risk of viral infection using an antiviral agent (CDV, BCV, and letermovir) [66].
**US2021000839A1** (Hadasit Medical Research Services and Development; Under examination)	A method of treating a viral infection (HSV and CMV) in a patient (newborn, a pregnant woman, and a transplant recipient) using a synergistic combination of an antiviral agent (BCV, CDV, valganciclovir, letermovir, and ganciclovir) and artemisone [67].
**WO2022072842A1** (Microbion Corporation; No national phase entry)	A method for treating osteomyelitis using a bismuth-thiol (BT) composition alone or in combination with an antimicrobial agent (BCV) [68].
**WO2021195236A1** (Microbion Corporation; No national phase entry)	A method for treating respiratory viral infection (viral pneumonia, viral bronchiolitis, and post-lung transplantation) using a bismuth-thiol (BT) composition alone or in combination with an antimicrobial agent (BCV) [69].
**WO2021186439A1** (Bar-Ilan University; No national phase entry)	A method of treating coronavirus infection using a combination of a macrolide (azithromycin) and a corticosteroid (desoxycortone), wherein this combination may optionally contain BCV [70].

## Data Availability

Not applicable.
